# Reducing surgical trays to cut both carbon emissions and costs in total knee arthroplasty

**DOI:** 10.2340/17453674.2025.43677

**Published:** 2025-05-27

**Authors:** Pim W VAN EGMOND, Paul LODDER

**Affiliations:** 1 Department of Orthopaedics, Elisabeth Tweesteden Hospital, Tilburg; 2 Department of Methodology and Statistics, Tilburg University, Tilburg; 3 Department of Medical and Clinical Psychology, Tilburg University, Tilburg; 4 Department of Education, Office of Science, Elisabeth Tweesteden Hospital, Tilburg, The Netherlands

## Abstract

**Background and purpose:**

Operating theatres are significant contributors to hospital waste and carbon emissions. In total knee arthroplasty (TKA), the number of surgical trays — and thus the carbon footprint — may be reduced by accurately estimating the prosthesis size preoperatively. We aimed to develop a predictive model to improve preoperative estimation of femoral prosthesis size and reduce the number of trays used in primary TKA.

**Methods:**

We retrospectively reviewed all primary TKA procedures performed between January 2012 and November 2022 at a single teaching hospital in the Netherlands. Using repeated hold-out cross-validation, we developed a prediction model based on routinely available demographic and anthropometric data to predict femoral component size. Rather than minimizing instruments per tray, our strategy focused on reducing the total number of trays. We used the created prediction model in combination with frequency data from our implanted TKAs to tailor surgical trays accordingly. We performed a post-hoc analysis to estimate the carbon emission cut and cost reduction.

**Results:**

The best-performing models utilized overlapping tray size ranges, with a practical limit of 3 sizes per tray. The final model predicted the appropriate size range with 97.4% accuracy. This enabled the elimination of 1 tray from the standard surgical setup, reducing total tray use by 11%.

**Conclusion:**

Accurate preoperative prediction of femoral prosthesis size facilitates surgical tray reconfiguration. We were able to reach an 11% reduction in total trays used with an estimated 1.03 kgCO_2_eq and a €29.6 cost reduction per reduced tray.

Climate change driven by greenhouse gas (GHG) emissions is increasingly recognized as a critical issue in healthcare, which contributes approximately 4.4% of global emissions—equivalent to the fifth largest emitter worldwide [1]. Among surgical specialties, orthopedic procedures—particularly total hip and knee arthroplasty (THA and TKA)—generate disproportionately high waste volumes, up to 60% more than other fields [2,3]. Recycling protocols and education programs could reduce the environmental impact of these procedures by as much as 75% [4]. Operating rooms are resource-intensive, with over 90% of their energy used for maintaining heating, ventilation, HVAC systems and powered equipment essential to orthopedic procedures. As such, the orthopedic community clearly has a responsibility to reduce both waste and energy consumption associated with surgical interventions [5].

A recent review identifies 5 key targets for reducing the environmental impact of orthopedic surgery: operating room waste, transportation emissions, manufacturing processes, anesthetic gases, and water use [6]. One actionable area is surgical tray optimization, which contributes significantly to carbon emissions through the number and size of trays, sterilization wrapping, and the energy required for sterilization [7]. Transitioning to reusable containers has been shown to reduce GHG emissions by 50–85% compared with single-use wrapping [7,8].

While many sustainability measures apply broadly across specialties, tray optimization is uniquely dependent on orthopedic expertise. Efforts should prioritize reducing the number of trays rather than minimizing instruments within them. Instruments should not be packaged separately unless doing so eliminates an entire tray, as separate processing increases emissions 2–3 times per item [9]. Instruments needed only in complex cases can be placed in optional trays, but their use must be balanced with practical considerations such as storage and surgeon preferences.

Estimating TKA implant size preoperatively offers a pathway to tray reduction. The approximate size of femoral and tibial components for TKA can be predicted using demographics and anthropometric measurements [10-13]. Digital templating is accurate and can help estimate implant size, reducing surgical sets [14]. However, not all centers have calibrated radiographs for preoperative templating. When available, templating is often performed shortly before surgery, limiting its effectiveness in ensuring the appropriate implants are prepared for planned cases. Additionally, if extra radiographs are required for templating TKA cases, this approach becomes less environmentally friendly. In contrast, patient data such as height, weight, BMI, sex, and side are readily available well in advance of scheduled procedures.

We aimed to reduce the number of surgical trays required for primary TKA by developing a predictive model to more accurately estimate femoral component size using preoperative patient data.

## Methods

### Study design

A retrospective review was performed of 6,698 sequential primary TKA procedures between January 2012 and November 2022 at a single teaching hospital in the Netherlands. In this period the PFC Sigma Total-Knee Replacement System (Depuy Synthes, Warsaw, IN, USA) was used. Primary TKA implant data of these procedures were collected from the Dutch Arthroplasty Register (LROI), which contains information on the patient height, weight, BMI, and sex, as well as all implant specifics.

### Patients and measurements

Only patients with the PFC Sigma Total-Knee Replacement System with complete implant, demographic, and anthropometric details were included in the study. Implant size dimensions were obtained from the manufacturer. The reporting guideline used was the STROBE checklist.

### Identifying possible factors in surgical tray reduction

The Deutsches Institut für Normung (DIN) has standardized the universal surgical tray size at 1 DIN (480 x 250 mm), though other sizes, such as 0.25 and 0.5 DIN, are also commonly used. We strive to reduce the number of trays or total tray size in DIN. Therefore, it is important to consider the space that instruments occupy on our surgical trays. We identified 3 categories of instruments in orthopedic surgical sets: universal, procedure-specific, and side/size/type-specific. Categorizing these instruments helps determine which can be reduced, and how (Table 1). In our TKA instrument sets, focusing on size-specific instruments offers greater reduction potential than side-specific ones. In TKA surgical trays, femoral instruments, which are size- and side-specific, take up significant space. Meanwhile, tibial instruments, which are only size-specific, occupy minimal space. Thus, we aim to reduce femoral instrumentation to maximize space savings.

**Table 1:** The 3 surgical instrument categories and the possible actions to reduce the number of instruments or surgical trays

Categories	Possible actions
*A: nonspecific* Remove instruments which are never used and unnecessary for acute perioperative solutions Choose between instruments with the same function Is it a generic tray? (i.e., is it used for different kind of procedures. Evaluate whether all procedures use a significant amount of the instruments Method: Actual instrument usage and expert recommendations [15]	
*B: procedure specific; not size/side/type specific* Choose between standard surgical options (i.e., intra- or extramedullary tibial referencing) Can instruments be reduced by creating consensus in the use of these instruments? Evaluate the frequencies of procedure specifics (i.e., patellar resurfacing or rarely used trial inserts, sawing guides) Method: Actual instrument usage and expert recommendations [15]	
*C: procedure specific; size/side/type specific* List all items in this category by size, side, or type to highlight which of these has the greatest possible reduction potential Side: side-specific trays Size: size-specific (ranges) trays, prediction model Type: type-specific trays (i.e., CR and PS trays) Consider a combination of side-/size- or type-specific trays	

CR = cruciate retaining; PS = posterior stabilized.

### Standard surgical trays for primary TKA

The tibial component comes in 7 sizes and is not side-specific, while the femoral component is available in 8 side-specific sizes with uneven increments between sizes (Table 2). All femoral sizes have cruciate-retaining (CR) and posterior-stabilized (PS) variants. Femoral finishing guides are size-specific but not side- or type-specific. PS femoral components require additional size-specific box sawing guides. Trial inserts for the 7 tibial sizes in CR and PS types of 8, 10, 12.5, and 15 mm are on our standard tray, though this does not cover all possible inserts of this prosthesis. There are 4 sizes for patellar resurfacing, which are not side- or type-specific.

**Table 2:** Anteroposterior (AP) and mediolateral (ML) tibial and femoral implant dimensions in millimeter by size

Size	Tibia AP/ML	Femur AP/ML
1.5	38/58	57/53
2	43/64	56/60
2.5	45/67	59/63
3	47/71	61/66
4	51/76	65/71
4 Narrow	–	65/68
5	55/83	69/73
6	59/89	74/78

Our standard TKA setup includes 7 DIN 1 trays: 2 with basic, non-prosthesis-specific instruments (including a burr and saw) and 5 with standard instruments for primary TKA. Of the 7 tibial sizes, 5 are on the standard tray; the smallest size must be requested in advance, and the largest is on a separate tray opened as needed. For the 8 femoral sizes, 5 are on the standard tray, with the smallest (1.5) by request and the largest on the same separate tray as the tibial component. Additionally, a narrower mediolateral option for size 4 femoral implants is available on a separate tray (Table 3).

**Table 3:** Number of prosthesis-specific instruments and surgical trays in which they are housed

Item	Standard TKA trays	PS tray	Size 6 tray (CR and PS)	Size 4 Narrow (CR and PS)
Surgical trays	5	1 additional	1 additional	1 additional
DIN^a^	1 each	1	1	0.5
Sawing guides				
femur	5	5	2	–
tibia	5	–	1	–
Trial prostheses				
femur (left/right)	10 (5/5)	5	2 (1/1)	2 (1/1)
extras	4 patella	screwdriver	–	–
femur adjustment jig^b^		1	1	1
Trial inserts				
sizes x thickness	5 x 4	5 x 4	1 x 4	–
options	CR	PS	PS and CR	–
Total prosthesis specific instruments on trays	44	31	14	3

CR = cruciate retaining; PS = posterior stabilized; TKA = total knee arthroplasty.

a DIN = The Deutsches Institut für Normung, standardized universal surgical tray size at 1 DIN = 480 x 250 mm.

b Femur adjustment jig: the “box” of the PS prosthesis can be mounted on a CR trial implant to transform it into a PS trial implant.

A CR TKA uses 5 prosthesis-specific trays. A size 4 Narrow femur prosthesis requires opening a separate 0.5 DIN tray, while a size 6 needs another 1 DIN tray. A PS TKA also requires an additional 1 DIN surgical tray. Most instruments are used for a size 6 PS TKA, involving a total of 9 DIN 1 trays of which 7 DIN 1 trays contain 89 prosthesis-specific instruments (Table 3).

### Statistics

Patient characteristics were described in terms of mean and standard deviation for continuous characteristics and in terms of frequencies and percentages for categorical characteristics. A multinomial logistic regression was used to estimate the association between femoral size and the patient characteristics sex, length, weight, BMI, and prosthesis side. Estimates were presented as odds ratios including 95% confidence interval (CI).

We built a model to predict femoral size preoperatively and assessed various prosthesis size tray combinations to find the tray scenario with the lowest probability of needing an additional surgical tray during TKA. We evaluated potential tray sets with and without overlapping sizes. For example, one set with overlapping sizes is: Set 1 (sizes 1.5 and 6), Set 2 (sizes 2, 2.5, and 3), and Set 3 (sizes 3, 4, and 5). The prediction model was built in RStudio (R Foundation for Statistical Computing, Vienna, Austria) and the R-script is available on this project’s open science framework page: https://osf.io/rc6zm/.

#### Step 1: Building the prediction model

Repeated hold-out cross-validation was used to minimize overfitting and enhance out-of-sample performance. The data was randomly split into an 80% training set and a 20% test set. We then fitted a multinomial logistic regression model to the training data, using femoral prosthesis size as the dependent variable and patient characteristics (sex, height, weight, BMI, and prosthesis side) as predictors. This approach was chosen over linear regression to ensure the predicted values matched actual prosthesis sizes.

#### Step 2: Predicting the optimal prosthesis tray

In the test set, we used the trained model’s estimated regression coefficients to predict each patient’s femoral prosthesis size based on their demographic and anthropometric characteristics. For each patient, the predicted size was used to identify the set most likely to contain their optimal prosthesis. If the predicted size was in 2 sets, we summed the predicted probabilities of that patient for each set and selected the 1 with the highest total probability.

#### Step 3: Estimating prediction accuracy

Steps 1 and 2 were repeated 500 times to average the predictive performance across multiple cross-validation runs. In step 3, we calculated the accuracy of each tray set by the proportion of patients whose actual prosthesis size was in the predicted set. Lastly, we assessed the number of additional surgical trays needed and the total number of size-specific surgical trays that had been opened because we predicted the wrong prosthesis size. These figures were expressed per 100 patients.

### Quantifying carbon emissions and cost reduction

We performed a post-hoc analysis to estimate the carbon emission cut and cost reduction of reducing a surgical tray in our TKA set.

### Funding, use of AI, and disclosures

The authors report no funding was received for this work. No artificial intelligence tools were used in the writing or analysis of this manuscript. The authors have no conflicts of interest to disclose. Complete disclosure of interest forms according to ICMJE are available on the article page, doi: 10.2340/17453674.2025.43677

## Results

### Patient and implant characteristics

**Figure 1:**
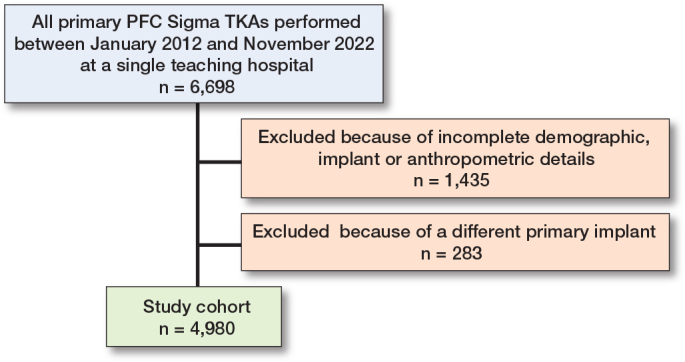
Flowchart of included cases. Only patients with complete implant, demographic, and anthropometric details were included. TKA = total knee arthroplasty.

Of the 6,698 initially retrieved primary TKAs, only patients with complete implant, demographic, and anthropometric details were included, resulting in 1,435 exclusions. An additional 283 cases were excluded due to the use of another primary implant than PFC Sigma (Figure 1). The 4,980 individuals in the study cohort were predominantly female (63%) with an average height of 169 cm (SD 9.7) and weight of 85kg (SD 15.6). A posterior-stabilized (PS) prosthesis was used in the majority of cases (68%), and patellar resurfacing was performed in most procedures (78%) (Table 4). Femoral sizes were symmetrically distributed, with sizes 3 and 4 being the most common.

**Table 4:** Characteristics of the 4,980 patients included in the sample and frequencies of size, side, and type of implanted TKA. Values are account (%) or mean (standard deviation [SD]) as specified

Category		
Sex	Male	1,826 (37)
	Female	3,154 (63)
Height, mean (SD)		168.6 (9.7)
Weight, mean (SD)		85.3 (15.6)
BMI, mean (SD)		30.1 (5.0)
Prosthesis side	Left	2,366 (48)
	Right	2,614 (52)
Femur type	CR	1,569 (32)
	PS	3,411 (68)
Femur size	1.5	2 (0.04)
	2.0	188 (3.8)
	2.5	749 (15)
	3.0	1,616 (32)
	4 Narrow	346 (6.9)
	4.0	1,130 (23)
	5.0	866 (17)
	6.0	83 (1.7)
Tibia size	1.5	2 (0.0)
	2.0	312 (6.3)
	2.5	1,186 (24)
	3.0	1,648 (33)
	4.0	1,325 (27)
	5.0	495 (9.9)
	6.0	12 (0.2)
Insert thickness, mm	8	2,413 (49)
	10	1,995 (40)
	12.5	479 (9.6)
	15	87 (1.7)
	17.5	4 (0.1)
	20	1 (0.0)
	22.5	1 (0.0)
Patellar resurfacing	Yes	3,902 (78)
	No	1,078 (22)

### Prediction model

In the predicting model of femoral size using sex, height, weight, BMI, and prosthesis side all predictors were statistically significant, supporting their inclusion in the model to accurately predict the optimal surgical tray composition (Table 5).

**Table 5:** Estimates of the multinomial logistic regression, predicting in the full sample (N = 4,980) the femoral size based on patient sex, height, weight, BMI and side. Estimates are presented as odds ratios (including 95% confidence interval) for a particular femoral size relative to the reference size 3

Femoral size	Sex	Height	Weight	BMI	Side
1.5	0.16 (0.16–0.16) ^a^	0.85 (0.80–0.91) ^a^	0.75 (0.57–0.98) ^a^	1.44 (0.96–2.17)	0.09 (0.09–0.09) ^a^
2.0	5.82 (0.73–46.7)	0.74 (0.73–0.75) ^a^	1.08 (1.04–1.11) ^a^	0.77 (0.71–0.84) ^a^	1.18 (0.85–1.63)
2.5	3.86 (1.62–5.05) ^a^	0.84 (0.83–0.84) ^a^	1.07 (1.05–1.11) ^a^	0.80 (0.77–0.84) ^a^	1.35 (1.12–1.62) ^a^
3.0 (Ref.)					
4.0	0.21 (0.17–0.27) ^a^	1.20 (1.20–1.21) ^a^	0.96 (0.94–0.97) ^a^	1.16 (1.11–1.21) ^a^	0.90 (0.76–1.06)
5.0	0.02 (0.01–0.03) ^a^	1.30 (1.29–1.30) ^a^	1.01 (0.96–1.09)	1.03 (0.96–1.09)	0.81 (0.63–1.03)
6.0	0.02 (0.00–0.12) ^a^	1.64 (1.62–1.65) ^a^	0.91 (0.87–0.94) ^a^	1.46 (1.29–1.65) ^a^	0.86 (0.52–1.43)

a P < 0.05.

To reduce the number of surgical trays in our TKA setup, we need to decrease the number of size-specific femoral components, as these instruments occupy the most space. Table 6 presents the options that have been investigated in the current analysis. The options vary in having either 2 or 3 femoral size ranges and in whether there is an overlap of a particular size in these options (Table 6). 2 outcome measures are shown in the table. First, the prediction accuracy was calculated by averaging across all cross-validation replications the proportion of patients who received a predicted set that contained their prosthesis size. Second, the number of surgical trays needed for each patient was determined by checking whether the predicted set contained the correct size. Sets with up to 3 femoral sizes required 1 surgical tray, while those with more required 2. If the prediction was incorrect, the correct sets surgical trays were added. For example, in Table 6, option B consists of 3 sets with 1 (2 sizes), 1 (2 sizes), and 2 (4 sizes) surgical trays. If a patient’s correct size is in set 3 but predicted to be in set 2, 1 surgical tray for set 2 and 2 for set 3 are needed, totaling 3 surgical trays. This process was repeated across patients, and the average surgical trays required per scenario were calculated per 100 patients.

**Table 6:** For 12 investigated tray scenarios, the prosthesis sizes included in each of the 2 or 3 sets, information on whether the tray sizes are allowed to overlap, and the accuracy calculated by averaging across all cross-validation replications the proportion of patients that received a predicted set that contained their prosthesis size, and the predicted number of size-specific surgical trays required during surgery (per 100 treated patients)

Tray scenario	Set 1	Set 2	Set 3	Overlap	Accuracy % (CI)	Required size-specific surgical trays
A	1.5 & 6	2 & 2.5	3 & 4 & 5	No	81.9 (78.5–85.2)	118
B	1.5 & 6	2 & 2.5	2.5 & 3 & 4 & 5	Yes	96.2 (94.6–97.9)	206
C	1.5 & 6	2 & 2.5 & 3	4 & 5	No	82.0 (78.7–85.4)	118
D	1.5 & 6	2 & 2.5 & 3	3 & 4 & 5	Yes	97.4 (96.1–98.8)	116
E	1.5 & 6	2 & 2.5 & 3 & 4	4 & 5	Yes	97.7 (96.4–99.0)	167
F	1.5 & 2 & 2.5	3 & 4 & 5 & 6	–	No	83.6 (80.3–86.8)	213
G	1.5 & 2 & 2.5	2.5 & 3 & 4 & 5 & 6	–	Yes	97.9 (96.7–99.2)	204
H	1.5 & 2 & 2.5 & 3	4 & 5 & 6	–	No	83.7 (80.5–87.0)	177
I	1.5 & 2 & 2.5 & 3	3 & 4 & 5 & 6	–	Yes	99.2 (98.4–99.9)	228
J	1.5 & 2 & 2.5 & 3 & 4	5 & 6	–	No	87.5 (84.6–90.4)	199
K	1.5 & 2 & 2.5 & 3 & 4	4 & 5 & 6	–	Yes	99.4 (98.7–100)	164
L	1.5 & 2 & 2.5 & 3 & 4 & 5 & 6	–	–	No	100 (100–100)	200

**Figure 2:**
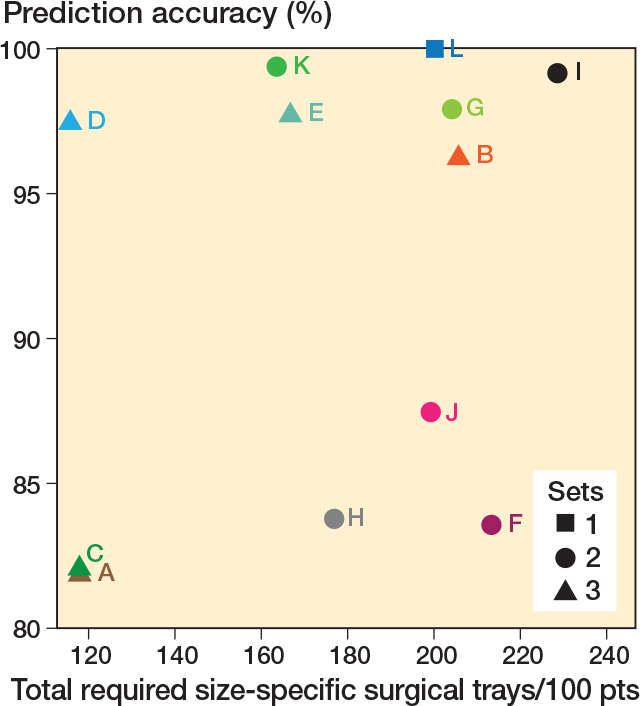
For each of the 12 investigated scenarios, the proportion of patients who received a predicted set that contained their prosthesis size (accuracy: y-axis) and the predicted number of size-specific surgical trays required during surgery (per 100 treated patients: x-axis). For tray scenarios A–L, see Table 6.

Figure 2 further illustrates these 2 outcome measures by plotting the prediction accuracy on the y-axis and the average number of size-specific surgical trays used on the x-axis. Squares, circles, and triangles indicate sets with 1, 2, or 3 surgical trays, respectively (Figure 2).

The results show that scenario L, containing all prosthesis sizes, had perfect prediction accuracy but did not reduce any surgical trays. Overall, sets with overlapping prosthesis sizes (B, D, E, G, I, K) demonstrated higher prediction accuracy, as shown by the non-overlapping CIs for those scenarios, than those without overlap (A, C, F, H, J) (see Table 4). Sets with more than 3 sizes could not fit in 1 surgical tray, making option E unsuitable. Thus, scenario D emerged as the best choice.

### Reducing a surgical tray

We subsequently adjusted our trays according to this best performing scenario (D) but, to be able to reduce a surgical tray, further adjustments were needed. The 15 mm trial insert and its gap balancer were used in only 1.7% of cases, and the patellar resurfacing instruments, though frequently used, took up a lot of space. Removing these created enough room to fit the 3 sizes of scenario D including the necessary PS instruments in 1 tray. The 15 mm trial inserts and gap balancer were placed in a 0.5 DIN tray to be opened only when needed, while the patellar resurfacing instruments were moved to a 1 DIN tray.

### New TKA setup and surgical trays needed

With our new TKA setup, we use 6 surgical trays instead of 7, with 4 of them being prosthesis-specific. We have reduced our standard setup by 2 femoral sizes, utilizing a prediction model to estimate the required size. We integrated this prediction model into our Electronic Patient File, allowing the estimated implant size to be determined well before surgery. If an overlapping size is predicted (size 3), the second most likely size determines whether the “small range” or “large range” will be prepared for the procedure. Additionally, we can now perform a PS TKA without needing an extra tray. However, we require an additional 1 DIN tray for patellar resurfacing and a 0.5 DIN tray for 15 mm insert trials. Due to a prediction accuracy of 97.4% (96.1–98.8), there are occasional instances where an extra tray is needed for incorrect size predictions. Nonetheless, if we had used our new setup, we reduced the total number of surgical trays needed by 11.2% (11.0–11.4%) across 4,980 cases, decreasing from 38,529 DIN to 34,212.5 DIN (Table 7).

**Table 7:** Number of surgical trays (in DINS) necessary for all 4,980 cases, before and after optimizing the surgical tray

Item	Standard set	Optimized set
Standard TKA	34,860	29,880
Outlier sizes		
Size 1.5	2	2
Size 4 Narrow	173	173
Size 6	83	83
15 mm insert	–	43.5
Wrong predicted size (CI)	–	129 (60–194)
Posterior stabilized femur type	3,411	–
Patellar resurfacing	–	3,902
Total amount of surgical trays required during surgery (CI)	38,529	34,213 (34,144–34,278)

### Carbon and cost reductions

A study in a Dutch hospital calculated the carbon footprint of a 1 DIN surgical tray at 2.14 kgCO_2_eq [7]. The use of reusable rigid sterilization containers, as employed in our practice, in place of single-use polypropylene (blue wrap) packaging, results in a 52% reduction in greenhouse gas (GHG) emissions—equating to an estimated 1.03 kgCO_2_eq reduction per tray eliminated [8].

Decontamination costs also vary across institutions and countries. An in-hospital sterilization service can impact costs significantly. A UK study reported a cost of €29.6 per tray [9]. Our hospital’s integrated sterilization services do not allow for precise cost calculation by specialty, but reducing decontamination needs clearly reduces overall costs.

## Discussion

We aimed to perform our standard TKA with fewer surgical trays but without compromising intraoperative surgical options or patient safety. To address this, we developed a prediction model for femoral prosthesis size using sex, height, weight, BMI, and prosthesis side. We showed that reorganizing the size-specific femoral instruments into 2 size ranges with an overlapping size had a 97.4% prediction accuracy. By adjusting our sets, we were able to reduce 11.2% of surgical trays used for primary TKA with an estimated 1.03 kgCO_2_eq and a €29.6 cost reduction per reduced tray.

A recent study in gynecological surgery demonstrated that integrating actual instrument usage data with expert recommendations in a computer-assisted model was the most efficient and patient-safe strategy for reducing surgical trays [15]. This method achieved a 36.7% reduction in instrument count and decreased the number of trays used, without any intraoperative instrument omissions. While effective for general surgical instruments, this approach is less applicable to orthopedic procedures, which often require size- and type-specific instrumentation. In our study, the reduction in trays was more modest at 11.2%. Further gains may be possible through greater surgical standardization, improved preoperative implant size prediction, and the development of more streamlined instrument sets.

Unlike waste management optimization, energy usage, and anesthesia improvements, this step requires action from orthopedic surgeons alone. The carbon footprint and cost reduction from eliminating a surgical tray depend on factors like the decontamination process, sterile barrier system, instrument set composition, and individually wrapped instrument needs [9]. A key factor is the proportion of low-carbon energy used, which varies by country and institution. Based on findings from a recent study in a Dutch hospital, which reported a carbon footprint of 2.14 kgCO_2_eq per DIN surgical tray, and accounting for the 52% reduction in greenhouse gas emissions achieved by our use of reusable rigid sterilization containers, we estimate a reduction of 1.03 kgCO_2_eq for each tray eliminated [7,8]. This is approximately equivalent to the emissions from producing 50 plastic 500 mL bottles for each TKA case and double that if disposable containers (blue wrap) are used. Optimal decontamination processes can further lower the carbon footprint by processing instruments in sets, utilizing decontamination test runs, maximizing machine loading, and minimizing standby time.

Similarly, decontamination costs vary across institutions and countries. An in-hospital sterilization service can impact costs significantly. A UK study reported a cost of €29.6 per tray and a recent study using a customized optimization model reduced the number of instruments on surgical trays by 42%, saving approximately €20,000 annually [9,16].

Although we have reduced the number of standard surgical trays for TKA, the set is still not particularly lean. Patient-specific instrumentation (PSI) could provide a leaner option but is not recommended for sustainability purposes. While Life Cycle Analysis (LCA) comparing reusable instrumentation with PSI is lacking, reusable instruments are generally more environmentally friendly. Typically, single-use instruments have greater carbon and financial costs than their reusable counterparts, with reductions in carbon footprint of 38–56% seen by switching to reusable equipment [17]. The environmental sustainability of robotic TKA surgeries has yet to be explored. However, robotic surgery in general has been consistently shown to increase environmental impact. A recent systematic review of laparoscopic surgeries found that robotic procedures resulted in 43.5% higher GHG emissions and 24% more waste production [18]. The authors concluded that the environmental impact of robotic surgery may not be justified by its clinical benefits. This insight should prompt us to weigh carefully the clinical advantages of robotic surgery against its potential environmental costs in our field.

### Limitations

While the prediction model may be applicable to other patient groups, it is tailored to the PFC Sigma Total-Knee Replacement System (Depuy Synthes, Warsaw, IN, USA), given size and increment variations between prostheses. However, our methodology is universal and can be adapted for all other TKA systems. In our practice, the smallest size was available only on request, potentially affecting prediction accuracy as surgeons might have opted for a size 2 when a size 1.5 would have been a better fit but was not available during surgery. In the LROI database, anthropometric measurements like height, weight, and BMI are optional. Many cases were excluded as some surgeons report only BMI.

The prediction model is just 1 aspect of reducing surgical trays. We also considered instrument frequency of use and tray space. Local preferences, tray sizes, and surgeon variability influence tray configurations at different hospitals using the same prosthesis. For example, as a teaching hospital, we include both intra- and extramedullary tibial guides for training. Intramedullary alignment generally requires less bulky instruments. Adopting it as the standard method could reduce instrument set sizes. We relocated the patellar resurfacing instruments to a separate tray, creating more space than separating PS instruments. This change was also prompted by a recent Dutch guideline update, which reduced our frequency of primary patellar resurfacing from 78% (as reported in this study) to 52% of cases. By placing these instruments on a separate tray, we further minimized the total number of trays required. This reduction is not reported in this study.

### Conclusion

It is possible to reduce surgical trays, and the environmental impact associated with TKA, if we make a little effort. In this study, we achieved an 11.2% reduction in surgical trays used for primary TKA, corresponding to an estimated carbon reduction of 1.03 kgCO_2_eq and cost savings of €29.6 per tray. Given that TKA is a high-volume, standardized procedure, even small reductions can lead to meaningful decreases in environmental burden and costs. It is like having your cake and eating it too.

## Notes

Handling co-editors: Marianne Westberg and Robin Christensen

*Acta* thanks Stephan Maximilian Röhrl and other anonymous reviewers for help with peer review of this manuscript.

## References

[1] Choi-Schagrin W. How hospitals fuel climate change. New York Times 2021, Nov 5.

[2] Phoon K M, Afzal I, Sochart D H, Asopa V, Gikas P, Kader D. Environmental sustainability in orthopaedic surgery. Bone Jt Open 2022; 3(8): 628-40. doi: 10.1302/2633-1462.38.BJO-2022-0067.R1. 35965477 PMC9422904

[3] Alam M M, Sujauddin M, Iqbal G M A, Huda S M S. Report: Healthcare waste characterization in Chittagong Medical College Hospital, Bangladesh. Waste Manag Res 2008; 26(3): 91–6. doi: 10.1177/0734242X07087661. 18649578

[4] Southorn T, Norrish A R, Gardner K, Baxandall R. Reducing the carbon footprint of the operating theatre: a multicentre quality improvement report. J Perioper Pract 2013; 23(6): 144-6. doi: 10.1177/175045891302300605. 23909168

[5] van Egmond P W, Meester R J, van Dijk C N. From big hands to green fingers: it is time for a change. J ISAKOS 2023; 8(4): 213-15. doi: 10.1016/j.jisako.2023.04.005. 37146690

[6] Saleh J R, Mitchell A, Kha S T, Outterson R, Choi A, Allen L, et al. The environmental impact of orthopaedic surgery. J Bone Joint Surg Am 2023; 105(1): 74-82. doi: 10.2106/JBJS.22.00548. 36574633

[7] Schmidt N, Sijm-Eeken M E, Langhout S A, Ruchtie L, Voorbraak F P, Sperna Weiland N H. A two-step approach to create and evaluate an optimization method for surgical instrument trays to reduce their environmental impact. Cleaner Environmental Systems 2023; 11: 1-14. doi: 10.1016/j.cesys.2023.100154.

[8] Friedericy H J, van Egmond C W, Vogtländer J G, van der Eijk A C, Jansen F W. Reducing the environmental impact of sterilization packaging for surgical instruments in the operating room: a comparative life cycle assessment of disposable versus reusable systems. Sustainability 2022; 14(1): 430. doi: 10.3390/su14010430.

[9] Rizan C, Lillywhite R, Reed M, Bhutta M F. Minimising carbon and financial costs of steam sterilisation and packaging of reusable surgical instruments. Br J Surg 2022; 109(2): 200-10. doi: 10.1093/bjs/znab406. 34849606 PMC10364739

[10] Blevins J L, Rao V, Chiu Y, Lyman S, Westrich G H. Predicting implant size in total knee arthroplasty using demographic variables: linear regression and Bayesian modelling. B Joint J 2020; 102(6_Supple_A): 85-90. doi: 10.1302/0301-620X.102B6.BJJ-2019-1620.R1. 32475285

[11] Kunze K N, Polce E M, Patel A, Courtney P M, Levine B R. Validation and performance of a machine-learning derived prediction guide for total knee arthroplasty component sizing. Arch Orthop Trauma Surg. 2021; 141(12): 2235-44. doi: 10.1007/s00402-021-04041-5. 34255175

[12] Naylor B H, Butler J T, Kuczynski B, Bohm A R, Scuderi G R. Can component size in total knee arthroplasty be predicted preoperatively? An analysis of patient characteristics. J Knee Surg 2021; 36(9): 965-70. doi: 10.1055/s-0042-1748902. 35820432

[13] Ostovar M, Jabalameli M, Bahaeddini M R, Bagherifard A, Bahardoust M, Askari A. Preoperative predictors of implant size in patients undergoing total knee arthroplasty: a retrospective cohort study. BMC Musculoskelet Disord 2023; 24(1): 650. doi: 10.1186/s12891-023-06785-0. 37582754 PMC10426207

[14] Cosendey K, Moerenhout K, Stanovici J, Jolles B M, Favre J. Intra- and inter-operator reliability of three-dimensional preoperative planning in total knee arthroplasty. Arch Orthop Trauma Surg 2024; 144(8): 3625-30. doi: 10.1007/s00402-024-05438-8. 39008074 PMC11417049

[15] van Nieuwenhuizen K E, van Trier T, Friedericy H J, Jansen F W, Dankelman J, van der Eijk A C. Optimising surgical instrument trays for sustainability and patient safety by combining actual instrument usage and expert recommendations. Sustainability 2024; 16(16): 953 doi: 10.3390/su16166953.

[16] Toor J, Bhangu A, Wolfstadt J, Bassi G, Chung S, Rampersaud R, et al. Optimizing the surgical instrument tray to immediately increase efficiency and lower costs in the operating room. Can J Surg 2022; 65(2): E275-E281. doi: 10.1503/cjs.022720. 35414528 PMC9007441

[17] Brighton and Sussex Medical School C for SHUHA on CC. Green Surgery: Reducing the environmental impact of surgical care [Internet]. London. Available from: https://ukhealthalliance.org/sustainable-healthcare/green-surgery-report/

[18] Papadopoulou A, Kumar N S, Vanhoestenberghe A, Francis N K. Environmental sustainability in robotic and laparoscopic surgery: systematic review. Br J Surg 2022; 109(10): 921-32. doi: 10.1093/bjs/znac191 35726503

